# Intrapulmonary Vascular Dilatation Evaluated by ^99m^Tc-MAA Scintigraphy and Its Association with Portal Hypertension in Schistosomiasis

**DOI:** 10.1371/journal.pntd.0002881

**Published:** 2014-06-26

**Authors:** Andréa Simone Siqueira de Queirós, Simone Cristina Soares Brandão, Ana Lúcia Coutinho Domingues, Liana Gonçalves Macedo, Maira Souto Ourem, Edmundo Pessoa Almeida Lopes

**Affiliations:** Universidade Federal de Pernambuco, Cidade Universitária, Recife, Pernambuco, Brazil; University of Maryland School of Medicine, United States of America

## Abstract

**Background:**

Portal hypertension is responsible for various complications in patients with schistosomiasis, among them intrapulmonary vascular dilations (IPVD). In cirrhotic patients the presence of IPVD is a sign of poor prognosis, but in patients with hepatosplenic schistosomiasis (HSS) there are no studies assessing the significance of this change. The aim of this study was to evaluate the occurrence of IPVD through ^99m^Tc-MAA scintigraphy in patients with HSS and its relationship with clinical, laboratory, endoscopic and ultrasound parameters.

**Methods:**

Cross-sectional study evaluating 51 patients with HSS. Patients were diagnosed with IPVD when the brain uptake of ^99m^Tc-MAA was higher than 6%. Subsequently, they were divided according to presence (G1) or absence (G2) of IPVD and variables were compared between groups.

**Results:**

Overall, 51 patients with mean age of 56±12 years were assessed. IPVD was observed in 31 patients (60%). There was no statistically significant differences between groups when clinical, laboratory and endoscopic parameters were compared. Regarding ultrasound parameters, the splenic vein diameter was smaller in G1 (0.9±0.3 cm) compared to G2 (1.2±0.4 cm), p = 0.029.

**Conclusion:**

In patients with HSS, the occurrence of IPVD by ^99m^Tc-MAA scintigraphy was high and was associated with lower splenic vein diameter, which can be a mechanism of vascular protection against portal hypertension. However, more studies are needed to determine the clinical significance of the early diagnosis and natural evolution of IPVD in this population.

## Author Summary

Intrapulmonary vascular dilatation (IPVD) is the key event in development of hepatopulmonary syndrome, an arterial oxygenation defect in patients with portal hypertension. IPVD diagnosis can be made by EchoDopplercardiography or ^99m^Tc-macroaggregated albumin scintigraphy (^99m^Tc-MAA), and ethiopatogeny is still unknown. In Northeastern Brazil, hepatosplenic schistossomiasis (HSS) is the main cause of portal hypertension. In cirrhotics, the presence of IPVD influences survival and candidacy for liver transplantation, however, in HSS patients, IPVD has been poorly studied, specially using lung perfusion scan with ^99m^Tc-MAA. Some authors believe that IPVD is common in HSS and in the existence of differences in indirect portal hypertension parameters between patients with and without IPVD. All patients were distributed into two groups according to presence or not of IPVD, and laboratorial, endoscopy and ultrasound tests were perfomed. Occurrence of IPVD was high and was associated with lower splenic vein diameter, which can be a vascular protection mechanism against portal hypertension status.

## Introduction

Hepatosplenic schistosomiasis (HSS) is characterized by the presence of liver fibrosis around the intrahepatic branches of the portal vein without damage to the hepatocyte synthesis ability [1]. It is estimated that schistosomiasis affects 2.5–6 million Brazilians [2] and about 10% of them develop the hepatosplenic form, which is the main cause of portal hypertension in Northeastern Brazil [3]. Portal hypertension is responsible for a number of complications in patients with schistosomiasis, like esophageal varices, gastrointestinal bleeding or intrapulmonary vascular dilatation (IPVD) [4], [5].

IPVD is the key event in the development of hepatopulmonary syndrome (HPS), when associated with alveolar-arterial difference of O_2_ >15 mmHg and liver disease with portal hypertension [6]. The natural history of IPVD has not been sufficiently understood, since its precise pathogenic mechanism remains unclear, but portal hypertension may be the initial stimulus for vasodilation [7]. In cirrhotic patients, the presence of IPVD is evidence of poor prognosis, especially when associated to HPS [4], but in HSS patients, there are no studies assessing the clinical significance and prognosis.

The method most widely used to diagnose IPVD is transthoracic Doppler echocardiography [6], [8], [9]; however, studies have suggested ^99m^Tc-macroaggregated albumin (^99m^ Tc-MAA) scintigraphy as alternative, for being sensitive, specific and because it quantifies the magnitude of IPVD [10]–[12]. In ^99m^Tc-MAA scintigraphy, radiolabeled particles, which usually have more than 20 µm in diameter, are injected and remain retained in the pulmonary vasculature, since capillaries measure 8 to 15 µm in diameter [12]. In the presence of IPVD, as in vasodilation, a fraction of these particles migrate to the systemic circulation, which can be quantified by radioactivity in the intra- and extrapulmonary circulation [6].

The aim of this study was to evaluate the occurrence of IPVD by ^99m^Tc-MAA scintigraphy and its association with clinical, laboratory, endoscopic and ultrasound parameters in patients with HSS.

## Materials and Methods

This is a descriptive, cross-sectional study involving intergroup comparisons. Fifty-one consecutive patients in treatment at the schistosomiasis outpatient Clinical Hospital – Federal University of Pernambuco (UFPE) with previous diagnosis of S. mansoni periportal fibrosis were evaluated between November 2010 and June 2012.

Patients of both sexes, aged over 18 years, with esophageal varices and HSS diagnosis were included. Diagnosis of schistosomiasis was previously obtained by means of epidemiological, and/or parasitological parameters and presence of periportal fibrosis, increased left lobe of the liver and ultrasound splenomegaly.

The following exclusion criteria were used: refusal to participate in the study, presence of chronic liver disease of other etiologies, previous pulmonary disease and/or intracardiac shunt, technical impossibility to perform the necessary tests and past splenectomy.

Patients were submitted to clinical evaluation and performed laboratory tests, endoscopy, abdominal ultrasound and ^99m^Tc-MAA scintigraphy. Peripheral blood was assessed for the following: albumin, bilirubin, AST, ALT, platelets, alkaline phosphatase and GGT using automated spectrometry and Cobas C501, Roche Diamond Diagnostics, USA. For measuring RNI, coagulometric automated method was used.

Endoscopic evaluation was performed using Olympus 100 videoendoscope using the criteria of Beppu, which ranks esophageal varices into fine, medium and large [13]. Ultrasound was performed by a single examiner with a Siemens Acuson X 150 device using a of 3.5 MHz convex transducer, assessing the following: diameter of the portal vein and splenic vein with normal values of up 1.2 and 0.9 cm respectively, longitudinal spleen diameter, with normal values of up to 12 cm, presence or absence of portal-systemic collateral circulation and periportal fibrosis pattern, classified as C/D/E/F according to the criteria of Niamey [14].

For ^99m^Tc-MAA scintigraphy, the patient was asked to stand in the upright position for 10 minutes and after this interval, 0.5 mL of ^99m^Tc-MAA was intravenously administered (average activity of 185 MBq of Technetium-99m with 300.000 MAA particles) in two minutes. Twenty minutes after tracer injection, the images were obtained in a gamma one-head camera (model STARCAM 3200, General Electric, California, USA), using a low-energy collimator for all purposes, photopeak of 140 Kiloelectronvolt (keV) and 20% window. Static images of chest and skull were obtained. Thorax images were obtained in anterior and posterior projections and skull images were obtained in lateral projections, with the patient in supine position.

For image acquisition, the detector was positioned approximately 10 cm away the patient's body, using a 128×128 matrix for five minutes. Subsequently, the images were analyzed by drawing regions of interest (ROIs) in skull and lungs. Patients were diagnosed with IPVD when the brain uptake of ^99m^Tc-MAA was higher than 6% [15].

The brain uptake was calculated using the following formula: (geometric mean of brain uptake/0.13)/(geometric mean of brain uptake/0.13)+geometric mean of pulmonary uptake [15]. According to the presence or absence of IPVD, patients were divided into two groups - G1 (positive IPVD) and G2 (negative IPVD) and the clinical (age, sex, history of gastrointestinal bleeding), laboratory, endoscopic and ultrasound features of each group were compared.

To compare quantitative variables, the Mann-Whitney U test was used, which does not need to assume data normality and has power efficiency around 95%. For qualitative variables, the chi-square test was used to verify if the group interferes in the variable being assessed. Differences between groups were considered statistically significant when p-value was <0.05.

### Ethic statement

The study was evaluated and approved by the Ethics Research Committee of the Center for Health Sciences - UFPE and all patients signed the informed consent form.

## Results

**Figure 1:**
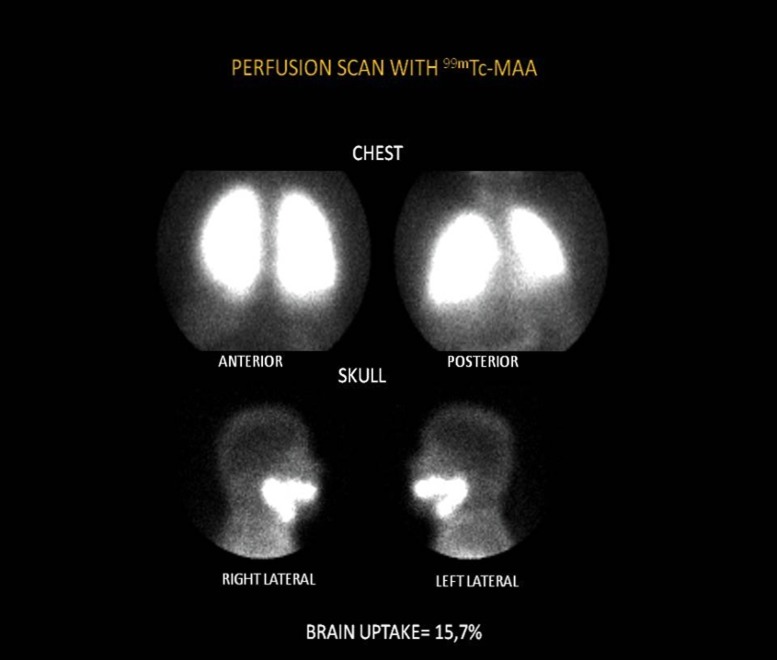
Anormal scintigraphy. Brain uptake = 15.7%.

**Figure 2:**
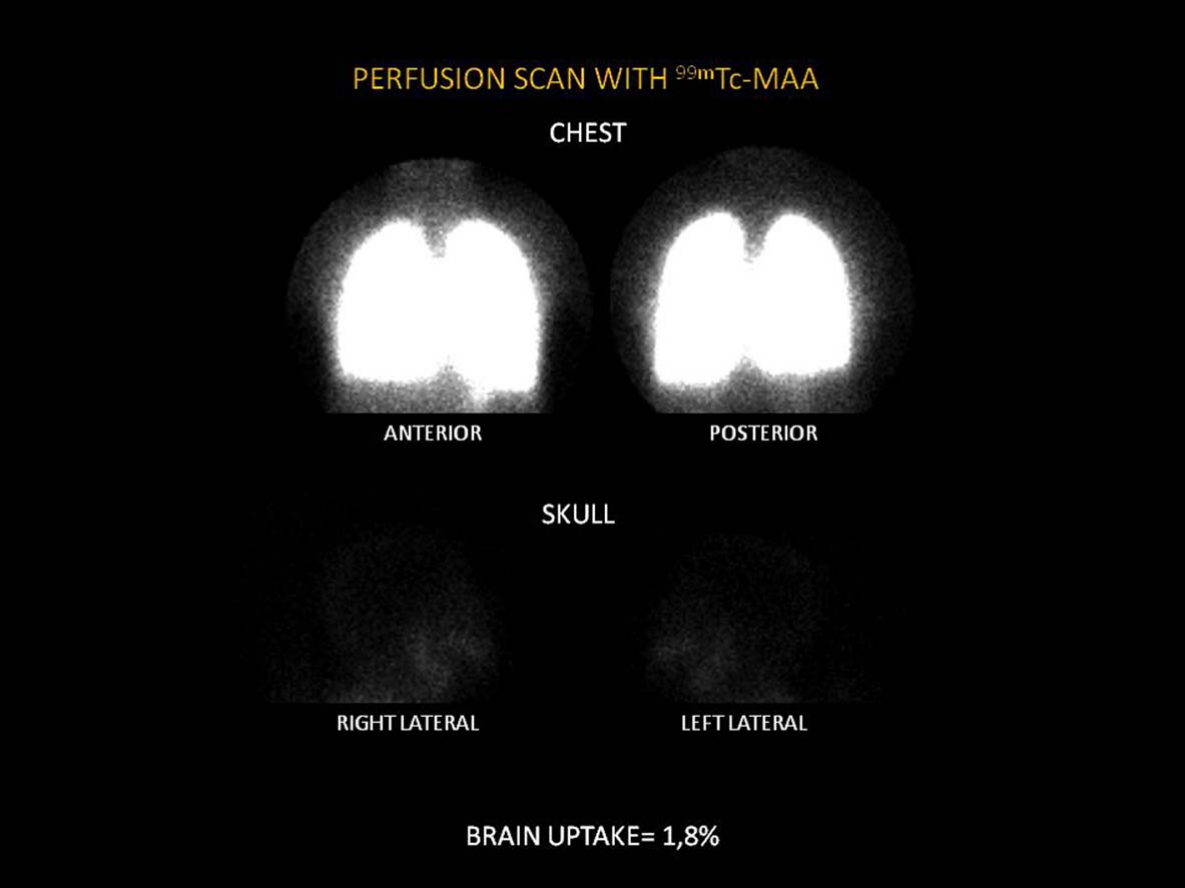
Normal scintigraphy. Brain uptake = 1.8%.

Overall, 51 patients were assessed, 33 females (64%) with mean age of 56±12 years. The presence of IPVD was observed in 31 patients (60%). Table 1 shows the clinical and laboratory parameters and Table 2 shows the endoscopic and ultrasound parameters. The average brain uptake was 9.2±2.6% in the group with IPVD and 3.8±1.2% in patients without IPVD (p<0.005). Figure 1 shows abnormal scintigraphy (brain uptake = 15.7%) and Figure 2 shows normal scintigraphy (brain uptake = 1.8%)

**Table 1:** Clinical and laboratory parameters.

Parameters	Total	G1	G2	p-value
**Clinical**				
Age (years)	55.8±13.2	56.7±13.9	54.6±12.2	0.573
Sex				
Female	33 (64.7%)	23 (74.2%)	10 (50%)	0.078
Male	18 (35.3%)	08 (25.8%)	10 (50%)	
Gastrointestinal bleeding				
No	30 (58.8%)	20 (63.3%)	10 (50%)	
Yes	21 (41.2%)	11 (36.7%)	10 (50%)	0.35
**Laboratory**				
Albumin (g/dL)	4.2±0.7	4.2±0.7	4.1±0.7	0.38
Bilirrubin (mg/dL)	1.1±0.5	1.1±0.5	1.2±0.5	0.13
RNI	1.2±0.2	1.2±0.2	1.2±0.2	0.15
AST (U/L)	38.2±18,5	36.5±17.9	37.1±15.9	0.72
ALP (U/L)	36.2±19.9	33.1±15.8	38.3±20.9	0.19
Platelets (×10^3^ p/mm3)	92±44	100±51	88±36	0.27
Alkaline phosphatase (UI/L)	116±52	107±46.3	126±57.5	0.19
γGT (UI/L)	120±82	96±67.6	141±105.2	0.19

RNI – Ratio Normalized International; AST – alanine aminotransferase; ALT – aspartate aminotrasnferase; γGT – γglutamil transferase.

**Table 2:** Endoscopics and ultrasounds parameters.

Parameters	Total	G1	G2	p-valor
**Endoscopics**				
Esophageal varices				
Grade I	39 (70%)	1 – 23 (73,3%)	12 (57,9%)	0.31
Grade II	08 (15%)	2 – 05 (16,7%)	03 (15,8%)	
Grade III	08 (15%)	3 – 03 (10,0%)	05 (26,3%)	
**Ultrasound**				
Portal vein(cm)	1.3±0.33	1.3±0.2	1.3±0.4	0.6
Splenic vein (cm)	1.00±0.3	0.9±0.3	1.2±0.4	0.029
Collateral circulation				
No	23 (45%)	11 (36%)	12 (60%)	0.14
Yes	28 (55%)	20 (64%)	08 (40%)	
Spleen longitudinal diameter (cm)	15.9±2.3	15.6±2.5	16.5±2.2	0.14
Fibrosis				0.73
C	12 (23%)	08 (25.8%)	04 (21.1%)	
D	31 (60%)	19 (61.3%)	12 (57.8%)	
E	08 (17%)	04 (12.9%)	04 (21.1%)	

When groups were compared, a higher percentage of women in the group with IPVD was observed (74.2% vs 50%), with p = .07. There were no statistically significant differences between groups regarding previous episodes of gastrointestinal bleeding or in relation to albumin (4.2±0.7 vs 4.1±0.7 g/dL), bilirubin (1.1±0.5 vs. 1.2±0.5 mg/dL), RNI (1.2±0.2 vs. 1.3±0.2), AST (37±18 vs 37±16 U/L) ALT (33±16 vs 38±21 U/L), and platelet values (100,000±51,000 vs. 88,000±36,000 per mm^3^).

When endoscopic parameters were evaluated, higher percentage of varices grades II and III in patients without IPVD was observed. Among ultrasound parameters, statistically significant differences were observed between groups when the splenic vein diameter was measured, with higher values for group without IPVD (0.9±0.3 in G1 vs 1.2±0.4 cm in G2, p = .029).

## Discussion

To the best of our knowledge, this is the first study to evaluate the IPVD frequency in HSS patients by ^99m^Tc-MAA scintigraphy and its association with indirect portal hypertension parameters. In this study, IPVD frequency was 60% and its presence was associated to lower splenic vein diameter. This interesting result may reflect a protective mechanism of the pulmonary vascular system against portal hypertension.

In cirrhotic patients, the occurrence of IPVD ranged from 13 to 47% [16], [17]. In a study with children with portal hypertension, Shabrawi et al. found nearly twice the frequency of IPVD when ^99m^Tc-MAA scintigraphy data were compared to those using echocardiography [10]. Using transthoracic Doppler echocardiography in patients with schistosomiasis already with gas exchange alterations (Da-Ao2>15 mmHg), Ferreira et al. found an IPVD frequency of 22% [8].

Studies comparing transthoracic Doppler echocardiography and ^99m^Tc-MAA scintigraphy showed conflicting results regarding sensitivity and specificity. This high frequency suggests greater sensitivity of ^99m^Tc-MAA scintigraphy, which is able to identify small dilations in the pulmonary vasculature even before the development of HPS [10], [18]. Furthermore, this study was carried out in an University Hospital and all patients had esophageal varices, suggesting that more severe patients are examined, which may be one of the factors for the high IPVD frequency found. However, there are no studies assessing the importance of early IPVD diagnosis or the factors that leads to the development of HPS from the emergence of vascular dilatation in HSS.

In cirrhotic patients, it is well established that the presence of IPVD associated with hypoxemia is related to a higher MELD score [19], [20] and a worse prognosis [4], [21]. In a study carried out with patients without abnormal liver function Eldridge et al. showed the emergence of IPVD during the performance of physical exercises with spontaneous resolution during rest. In this group, the formation of vascular dilatation may be a defense mechanism to increased vascular pressure and blood flow in the pulmonary circulation [22]. In patients with schistosomiasis, the clinical relevance of the presence of IPVD is not well defined. In this study, no differences in the parameters that evaluated liver function among patients with and without IPVD were observed.

Aller et al showed that in cirrhotic males with IPVD, the serum levels of female sex hormones are higher than in patients without IPVD [23]. In patients with schistosomiasis, as there is no significant change in liver function, there should be no difference in the progesterone and estradiol levels in male patients compared to normal individuals. The high IPVD frequency in female patients found in this study suggests that these hormones may participate in the pathogenesis of the formation of this change, with vasodilatory effect, but further studies are needed to evaluate this association.

In patients with HSS, the obstruction to the hepatic blood flow caused by periportal fibrosis leads to increased pressure in the portal venous system [24]. The changes caused by portal hypertension are among the leading causes of morbidity and mortality in patients with schistosomiasis [25]. Formation of portal-systemic collateral circulation and increased portal and splenic vein diameter are the main changes occurring in the portal venous system of patients with schistosomiasis [1], [26].

This study demonstrates that patients with schistosomiasis and IPVD had smaller splenic vein diameters, suggesting that vascular dilation would be a mechanism to reduce pressure in the portal territory. Moreover, the group with IPVD showed a greater proportion of patients with collateral circulation (64% vs 40%), lower average longitudinal spleen diameter (15.6 cm×16.5 cm) and high platelet levels (100×10^3^ vs 88×10^3^) although was not statistically significant. It was also found that the group with IPVD had a lower proportion of patients with varices of medium and large diameter, suggesting that this group presents lower portal pressure levels.

The number of patients included in this study may not have been sufficient to demonstrate the most significant differences in indirect markers of portal hypertension between groups with and without IPVD. Moreover, it is necessary to investigate whether these findings are also observed in individuals with portal hypertension of other etiologies. Our team is conducting another study evaluating the IPVD frequency in patients with chronic liver disease of mixed etiology. Further studies should be carried out in other regions and centers for comparison purposes.

### Conclusion

In patients with HSS, the occurrence of IPVD evaluated by ^99m^Tc-MAA scintigraphy was high and associated with lower splenic vein diameter, which can be a mechanism of vascular protection against portal hypertension. However, more studies are needed to determine the clinical significance of the early diagnosis and natural evolution of IPVD in this population.
